# Prior context in audition informs binding and shapes simple features

**DOI:** 10.1038/ncomms15027

**Published:** 2017-04-20

**Authors:** Claire Chambers, Sahar Akram, Vincent Adam, Claire Pelofi, Maneesh Sahani, Shihab Shamma, Daniel Pressnitzer

**Affiliations:** 1 Laboratoire des Systèmes Perceptifs, CNRS UMR 8248, Paris 75005, France; 2 Département d'Etudes Cognitives, École Normale Supérieure (ENS), PSL Research University, Paris 75005, France; 3 Department of Physical Medicine and Rehabilitation, Northwestern University and Rehabilitation Institute of Chicago, Chicago, Illinois 60611, USA; 4 Electrical and Computer Engineering & Institute for Systems Research, University of Maryland, College Park, Maryland 20742, USA; 5 Gatsby Computational Neuroscience Unit, University College London, London WC1E 6BT, UK

## Abstract

A perceptual phenomenon is reported, whereby prior acoustic context has a large, rapid and long-lasting effect on a basic auditory judgement. Pairs of tones were devised to include ambiguous transitions between frequency components, such that listeners were equally likely to report an upward or downward ‘pitch' shift between tones. We show that presenting context tones before the ambiguous pair almost fully determines the perceived direction of shift. The context effect generalizes to a wide range of temporal and spectral scales, encompassing the characteristics of most realistic auditory scenes. Magnetoencephalographic recordings show that a relative reduction in neural responsivity is correlated to the behavioural effect. Finally, a computational model reproduces behavioural results, by implementing a simple constraint of continuity for binding successive sounds in a probabilistic manner. Contextual processing, mediated by ubiquitous neural mechanisms such as adaptation, may be crucial to track complex sound sources over time.

## Abstract (web summary)

Perception can be swayed by prior context. Here the authors report an auditory illusion in which sounds with ambiguous pitch shifts are perceived as shifting upward or downward based on the preceding contextual sounds, explore the neural correlates, and propose a probabilistic model based on temporal binding.

Perception guides behaviour through information accrued by the senses. However, at any given moment, the information encoded by our sensory receptors is never enough to specify exactly the state of the outside world^1^,^2^. Resolving some degree of intrinsic ambiguity is, therefore, a crucial part of perceptual processing. One hypothesis is that humans use prior context to disambiguate current sensory information^3^,^4^. Prior context is likely to be informative, as physical objects tend to have persistence over time: in the case of hearing, it is unlikely for instance that someone's voice will jump over several octaves mid-sentence. Here, we report behavioural, neural and computational evidence showing that human auditory perception seamlessly binds prior context into current perceptual decisions, and that this influence is exerted over simple features over a wide range of sound parameters.

Contextual effects of prior information have been extensively documented in visual perception. Some of these context effects are ‘contrastive', as when the prolonged presentation of a motion stimulus in one direction creates the subsequent perception of motion in the opposite direction in a stationary stimulus^5^. Other context effects may be ‘attractive', as when a brief motion stimulus biases a subsequent ambiguous stimulus towards the same perceived direction of motion^6^. These two kinds of context effect are thought to provide distinct functional benefits: to maximize information transfer in the case of contrastive effects, and to stabilize perception over time in the case of attractive effects^7^,^8^.

In the auditory modality, context effects have been established in the case of speech perception, and most of these effects have been contrastive. In an influential series of experiments^9^, it was found that the perception of formants and thus vowel identity can be changed through brief exposure to a speech context containing shifted formants. This was interpreted as evidence of speaker normalization through contrast enhancement^9^ and viewed as a compensation for motor processes specific to speech^10^. However, generic auditory context effects have also been reported: contrast enhancement can be obtained with non-speech contexts^11^,^12^,^13^ or non-speech targets^14^,^15^. Thus, both speech-specific and auditory-generic processes may be involved in the contrastive context effects prevalent in speech^16^,^17^.

Contrastive context effects have also been reported in the perception of simple auditory features. The subjective location of a sound can be shifted away from that of a preceding context^18^,^19^; the prolonged presentation of amplitude modulation can elevate subsequent modulation detection thresholds^20^; the prolonged presentation of frequency shifts in spectral peaks or troughs produces a negative ‘afterimage' for the perceived spectral motion of subsequent similar sounds^21^. More recently, it has been demonstrated that prolonged exposure was not always necessary: even very brief contexts lasting as little as 100 ms in some cases can lead to contrastive shifts in the perception of spectral motion^22^,^23^.

There are comparatively fewer instances of attractive contextual effects in auditory perception. In the perception of speech, hysteresis has been found in the report of ambiguous words^24^. In the perception of frequency and pitch, regression to the mean has been reported for successive pitch judgements^25^. When ambiguity is added to pitch judgements, hysteresis has been observed, one of the hallmarks of attractive effects^26^,^27^. In auditory scene analysis, finally, the perceptual organization of ambiguous tone sequences is biased towards prior percepts^28^. Other contextual effects in auditory scene analysis may also be considered as attractive, such as when a component of a complex tone is captured by the preceding context to change the perception of vowel formants^29^.

Here, we report a novel effect of prior context on auditory perception. The context effect we discovered impacts a basic auditory feature, the perception of spectral motion, or frequency shifts. However, it is not based on prior exposure to this specific feature—rather, it reflects the temporal binding of successive frequency components. The effect is therefore not easily construed as contrastive nor attractive, but its functional consequence is to stabilize perceptual organization, like attractive effects^8^. The context effect is also unusually salient. Before reading further, a demonstration can be experienced with the Supplementary sound examples or online: http://audition.ens.fr/dp/illusion/

## Results

### Ambiguous frequency shifts

**Figure 1:**
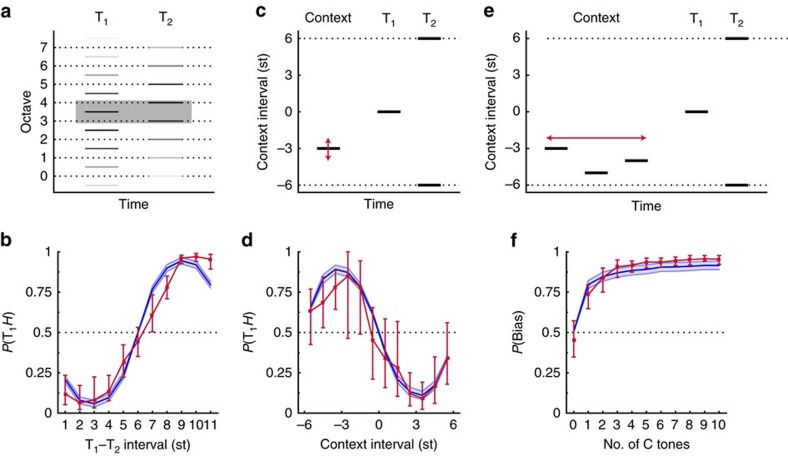
Ambiguous test pairs and context effects. (**a**) Schematic spectrogram of the T_1_–T_2_ test pair (amplitude coded as grey scale). T_1_ and T_2_ are ‘Shepard tones' with octave-related components. For the interval of half-an-octave (6 st) represented here, all components of T_1_ are exactly halfway in between two components of T_2_ on a log-frequency scale. (**b**) Perceptual judgements without context. The proportion of ‘T_1_ higher' responses, *P*(T_1_*H*), is plotted as a function of interval between T_1_ and T_2_ (*n*=11 listeners). Here and in all subsequent figures, error bars show 95% confidence intervals of the mean. The solid blue line shows the predictions of a probabilistic model fitted to the data (see text), with the blue shaded area showing 95% confidence intervals. The dotted line at 0.5 indicates the point of subjective indifference, with as many ‘up' and ‘down' responses for the same physical stimulus. (**c**) Example trial with a single context tone preceding the ambiguous test pair. For clarity, the illustration is restricted to a one-octave range (grey patch of **a**) but actual stimuli included many frequency components, with the same arrangement in all audible octaves. (**d**) The *P*(T_1_*H*) is shown as a function of the interval between Context and T_1_ (*n*=11 listeners). Without context effects, all responses would be at 0.5. (**e**) Example trial with multiple context tones. Listeners reported the perceived shift between T_1_ and T_2_ only. (**f**) The proportion of reporting a shift encompassing the frequency region of the context tones, *P*(Bias), is shown as a function of the number of context tones (*n*=11 listeners). In the baseline condition with no context tone, *P*(T_1_*H*) is displayed.

We used ambiguous sounds, as we hypothesized that these stimuli should be maximally sensitive to context effects^30^,^31^ Shepard tones were used^32^, consisting of many pure tones with octave relationships and covering all audible octaves (Fig. 1a). As a baseline, two Shepard tones (T_1_ and T_2_) were presented in close succession without prior context. The frequency interval between T_1_ and T_2_ was varied randomly across trials. We also introduced random intertrial sequences (see Methods) to minimize carryover effects across trials. Listeners reported which tone, T_1_ or T_2_, was higher in pitch.

Replicating previous findings^32^, listeners tended to report the direction of pitch shift corresponding to the shorter log-frequency distance between successive components (Fig. 1b). The ambiguous case occurred when the T_1_–T_2_ interval was half an octave (six semitones): there was no shorter path favouring either upward or downward shifts. Accordingly, perceptual reports were evenly split between ‘up' and ‘down' responses^31^,^32^,^33^. Strong idiosyncratic biases across listeners have been reported previously for such ambiguous stimuli^31^, but our randomization of absolute frequency across trials aimed at cancelling out such biases. Indeed, we verified that each individual participant provided ‘up' and ‘down' responses, in reasonably matched proportions (individual *P*(T_1_*H*)=0.45±0.26, median±interquartile range).

### Prior acoustic context resolves perceptual ambiguity

We then introduced an acoustic context before the pitch-shift judgements. A single context tone, also a Shepard tone, was played immediately before the test pair, T_1_–T_2_ (Fig. 1b). The T_1_–T_2_ interval was fixed at a half-octave, the ambiguous case. If context did not matter, responses should be evenly split between ‘up' and ‘down'.

In contrast, we found a strong influence of the context tone on perceptual reports. Listeners tended to report the shift encompassing the frequency components of the context tone, with maximal efficiency for a context tone located halfway in between T_1_ and T_2_ (Fig. 1d, Supplementary Table 1). This was confirmed by statistical analyses. Since the data did not always meet the requirements for parametric tests due to heteroscedasticity and lack of normality of the residuals, we performed nonparametric Kruskal–Wallis tests for all experiments. Permutation tests were used to estimate a *P* value from the *H* statistics (Supplementary Methods). In this first experiment, we observed a main effect of context interval on perceptual judgements (Kruskal–Wallis test, *H*(11)=57.70, *P*<0.0001, *N*=11).

In the same experiment, the number of context tones was randomly varied between 0 and 10 context tones. Context tones were randomly drawn, uniformly, from one of two half-octave frequency regions: only positive intervals or only negative intervals relative to T_1_ (Fig. 1e). We summarized the perceptual responses by computing *P*(Bias), the proportion of time listeners responded with a pitch shift consistent with a bias in the direction expected from Fig. 1d. A *P*(Bias) of 1 would correspond to listeners always reporting pitch shifts encompassing the frequency region of the context tones, whereas a *P*(Bias) of 0 would correspond to listeners always reporting the opposite direction of pitch shift. An absence of context effect, that is, a response probability unaffected by the context, would correspond to *P*(Bias) of 0.5.

The strength of the context effect increased with the number of context tones (Fig. 1f). The effect of context was confirmed by a Kruskal–Wallis test, which found a main effect of the number of context tones on *P*(Bias), *H*(9)=32.56, *P*<0.0001, *N*=11 (degrees of freedom are reduced because the value for the condition where no context was presented is plotted in the figure as baseline, but it was not included in the test). The *P*(Bias) statistics was further tested against the chance value of 0.5 by using bootstrapped confidence intervals, Bonferroni-corrected for the number of comparisons. This showed a significant effect of context in all cases, from 1 to 10 context tones (Supplementary Table 2). Remarkably, after about five context tones, nearly 100% of perceptual reports were consistent with the preceding context. Thus, for the exact same ambiguous test pair, listeners went from randomly reporting upward or downward shifts to almost invariably reporting the same direction of shift when context was added.

It is important to note that the sequence of ‘up' and ‘down' pitch shifts during the context was randomly varied within a trial and also across trials (see Fig. 1e for instance). The context therefore did not adapt a certain direction of shift. Rather, the relevant experimental variable was the frequency content of the context relative to the test. This resolves previously conflicting reports concerning contextual processing with Shepard tones. Attempts to adapt-out the direction of frequency shifts only resulted in weak and unreliable contrastive context effects^34^,^35^. In contrast, studies that, retrospectively, can be understood as having manipulated the frequency relationship between context and test, found strong context effects^26^,^27^,^33^.

### Large sample size and variable listening conditions

To test for the robustness of the context effect, we conducted an online experiment, with an unscreened sample of *N*=100 participants. Participants first performed ‘pitch-test' trials to evaluate their ability to identify one-semitone pitch shifts. Then, in the main part of the experiment, we used the same task as before, with 10 context tones.

A strong context effect was found for an overwhelming majority of participants (Supplementary Fig. 1). The effect was comparable in magnitude to the laboratory-controlled experiment. Interestingly, a subset of participants displayed poor performance on the pitch-test trials, but they still exhibited a strong contextual bias. This suggests that the contextual effect was not dependent on the participants' ability to explicitly identify small pitch shifts^36^.

### Generalization over timescales

We next examined whether the context effect we observed could generalize to longer and shorter timescales. In the case of the biasing of ambiguous visual motion^6^, it has been observed that attractive effects occurred for short timescales, whereas contrastive effects occurred for longer timescales. In the case of speech, contrastive effects have been found both for short timescales (tens to hundreds of milliseconds)^13^,^37^,^38^ and for longer timescales (a few seconds corresponding to the duration of sentences^39^,^40^,^41^). It is thus possible that our auditory context effect is maintained, reverses or disappears, depending on the timescale of the stimulus.

**Figure 2:**
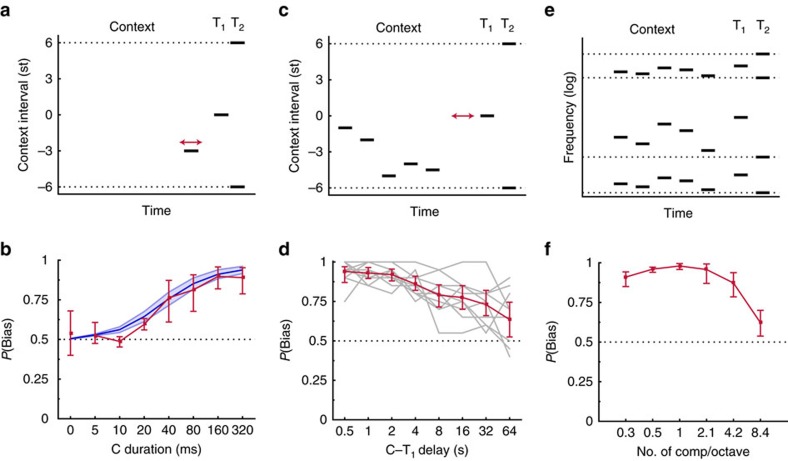
Generalization over time and frequency scales. (**a**) Example trial testing the effect of the duration of a single context tone. (**b**) The mean *P*(Bias) is displayed as a function of the context-tone duration (*n*=10 listeners). In the baseline condition without a context tone, *P*(T_1_*H*) is displayed. The solid blue line shows the predictions of a probabilistic model fitted to the data, with the blue shaded area showing 95% confidence intervals. (**c**) Example trial testing the effect of a silent gap between context and test. (**d**) The mean *P*(Bias) is displayed as a function of the Context-T_1_ silent gap (*n*=10 listeners). Individual listeners are shown by grey lines. (**e**) Example trial testing random spectra. Components of T_2_ were distributed randomly, but each exactly in-between two components of T_1_. The context-tone components were restricted to favour only one possible direction of shift between T_1_ and T_2_. Dotted lines represent one frequency ‘cycle' of the stimulus, they would be equally spaced at one octave for Shepard tones. (**f**) The mean *P*(Bias) is shown as a function of the number of components per octave (*n*=10 listeners).

We first measured how fast the context effect could be established. A single context tone was used and its duration was varied between 5 and 320 ms, with the duration of the test tones T_1_ and T_2_ maintained at 125 ms (Fig. 2a). The same direction of perceptual bias was observed over a broad range of context-tone durations (Fig. 2b). The bias increased with context duration and saturated for context durations of 160–320 ms. A Kruskal–Wallis test confirmed a main effect of duration on *P*(Bias), *H*(6)=43.34, *P*<0.0001, *N*=10. The *P*(Bias) was further tested against the chance value of 0.5 at each duration. This showed a significant context effect from and above durations of 20 ms, using a conservative Bonferroni correction (Supplementary Table 3).

We also addressed the complementary question of how long the bias persisted, once established. Five context tones were presented, each 125-ms long, followed by a silent gap and then a test pair (Fig. 2c). The gap was varied between 0.5 s to 64 s, during which attention was not controlled. Predictably, the bias decreased with increasing gap duration (Fig. 2d). A Kruskal–Wallis test confirmed a main effect of the Context–Test gap duration on *P*(Bias), *H*(7)=37.62, *P*<0.0001, *N*=10. The *P*(Bias) statistics was further tested against the chance value of 0.5 for each gap duration. This showed a significant context effect up to and including gap durations of 32 s, again using a conservative Bonferroni correction (Supplementary Table 4). In the individual data, for some listeners, there was in fact very little decrease in bias between 0.5 and 64 s.

The present context effects covers a wide range of timescales: it is induced with a context as short as 20 ms but persists over interruptions up to 64 s for some listeners. For comparison, the contrastive effects in speech have been demonstrated for a context as brief as about 100 ms (refs 13,37) or ever briefer when one considers the identity of a vowel surrounded by syllable-initial and syllable-final consonants, each lasting only 20 ms (ref. 38). The persistence of the speech contrastive effect has been observed for interruptions of up to 10 s (refs 9,12). Short time constants have also been found for the spectral motion after-effect, which can be observed with inducers as brief as 100 ms (refs 21,22). To the best of our knowledge, long persistence of auditory context effects on the order of 1 min has not yet been reported. Such long time constants are in fact reminiscent of what has been termed ‘storage' in the study of visual aftereffects^42^, but not demonstrated yet in audition. The present auditory context effect thus covered an unusually broad range of temporal parameters.

Importantly, that context effects can be both rapidly established and persist for a long time show that their underlying mechanisms may operate in most everyday auditory scenes. For instance, the median duration of short segmental cues in natural speech, such as unstressed vowels and consonants, is ∼70 ms (ref. 43). This is longer than the minimal duration of 20 ms required for observing context effects. Conversely, a persistence of 32 s as we observed is enough to accommodate prosodic cues^43^ and even pauses between sentences. Similar timescales may be found for music^44^. The timescales covered by our context effects thus encompass those typical of speech and music.

### Generalization over frequency scales

So far, we used highly constrained Shepard tones as a tool to probe and highlight contextual processing, but it is important to test whether the effects we observed can generalize to other types of sounds. We therefore used sounds with as few constraints as possible with respect to their frequency content: sounds with completely random spectra. Moreover, the density of the random spectra was systematically varied, from very dense with ∼8 tones per octave, to very sparse with ∼0.3 tones per octave. The only constraint we preserved was to enforce ambiguous frequency shifts in the final test pair, as ambiguity in this test pair was germane to our paradigm being sensitive to context effects.

The stimuli are illustrated in Fig. 2e. Context sequences were constructed so that frequency components of the context would be expected to favour only one direction of shift within the test pair. Results showed that all random-spectra stimuli produced a large bias, from the sparsest to the densest, with a decrease for the densest condition (Fig. 2f). A Kruskal–Wallis test revealed a significant main effect of density on *P*(Bias), *H*(5)=31.57, *P*<0.0001, *N*=10. The *P*(Bias) statistics was further tested against the chance value of 0.5. This showed a significant context effect for all spectral densities after Bonferroni correction, although for the densest case the confidence interval almost overlaps with 0.5 (Supplementary Table 5).

The context effect was observed for random-spectra stimuli, which were completely different from the Shepard tones used so far. Moreover, the effect was robust when tested over different frequency scales, from very sparse sounds to very dense sounds. It is likely that in the densest case of eight components per octave, which showed a decline of the magnitude of effect, the overall spectral pattern was starting to become unresolved within auditory cortex^45^. The only limit for the context effect in terms of frequency content thus seems to be to stimulate non-overlapping frequency regions. A similar robustness to spectral scale was found in the standard speech contrast effect^39^.

This generalization to arbitrary sounds again suggests that the mechanisms underlying the context effect could apply to typical natural auditory scenes. Given that the context effect were just as strong for random spectra as for Shepard tones, the spectral shape of the sounds should not matter at all. The limiting factor seems to be spectral density, and our results show that at least the first eight harmonics of any periodic sound, such as the voiced parts of speech or musical instruments sounds, would fall within the existence region of the context effect.

### Neural correlates of the perceptual bias

We next used brain imaging to probe the underlying neural mechanisms of the perceptual bias. A minimal assumption is that such mechanisms have to maintain a neural trace of the context sequence until the appearance of the test pair^46^,^47^. This trace could take many forms: a sustained increase in the firing rate of some neural pool, a sustained decrease in firing rate of such pool, or it could be latent, for instance expressed as a modulation of functional connectivity between neurons^48^. Moreover, the trace could be located at a decisional level^49^, or within sensory regions^50^,^51^, or both.

**Figure 3:**
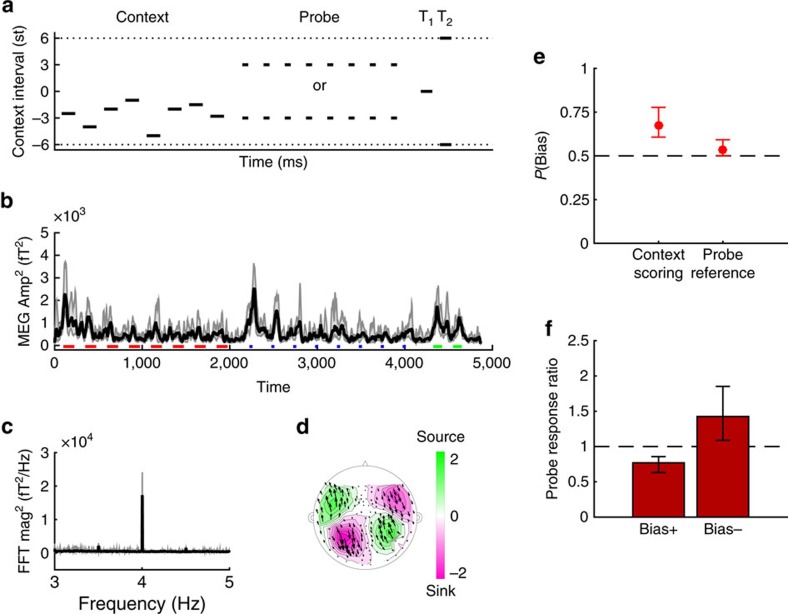
MEG recordings. (**a**) The behavioural paradigm was as before except that Probe tones were introduced between the Context and Test. The Probe-tone frequency was either the centre of the frequency region of the context (‘Context+') or the centre of the opposite frequency region (‘Context−'). Trials were coded as ‘Bias+' if listeners reported the expected pitch shift encompassing the frequency region of the context, or ‘Bias−' if listeners reported the opposite pitch shift. (**b**) Time course of the MEG response. The MEG sensor signals were filtered between 2 and 120 Hz, squared and then averaged for the 50 auditory channels for each participant. Here and in all panels the shaded area or error bars indicate 95% confidence intervals of the mean across participants. (**c**) Probe-tone response. A fast Fourier transform was performed on the concatenated MEG responses during the probe sequence over all trials, for each participant. For display purposes, the result was normalized for each participant by baseline activity over 3–5 Hz, excluding the frequency bin at 4 Hz. (**d**) The topography of the 4 Hz response during the probe sequence. The complex responses from the FFT analysis are plotted for each sensor, with the arrows representing the magnitude and phase value of the 4-Hz component. Contours are projections on lines of constant phase (see Supplementary Methods). (**e**) Behavioural results during the MEG recordings. The bias caused by context tones is of the same magnitude as for previous experiments, whereas the bias caused by the Probe sequence is weak, with confidence intervals for *P*(Bias) overlapping with 0.5. (**f**) MEG correlate of the behavioural bias. A PRR was defined as the 4-Hz probe response for Context+ trials, divided by the 4-Hz probe for Context− trials. A PRR value lower than one is observed for Bias+ trials, indicating a reduced responsivity in the frequency region of the context when the context was effective in producing a behavioural bias. The reverse pattern is observed, for identical stimuli, when the behavioural bias is in the opposite direction.

To test for some of these possibilities, we recorded magnetoencephalographic (MEG) activity while listeners performed the behavioural task. As a latent trace or even an endogenous sustained trace may not be detectable by time-locked MEG-evoked potentials, we introduced a series of short ‘probe' tones between the context sequence and test pair (Fig. 3a). Probe tones were Shepard tones located either at the centre of the context frequency region, where the contextual neural trace should be strongest because this was the condition where the behavioural bias was the most pronounced (see Fig. 1d), or located at the centre of the opposite frequency region, which should be relatively unaffected by context. We aimed at quantifying any modulation of activity of the probe tones that correlated with behavioural responses, all other acoustic characteristics of the stimuli being equal. This approach targets perceptually-relevant correlates, and excludes all other stimulus-related but potentially perceptually-irrevelant changes in the MEG response (ref 47)^47^.

Before the MEG experiment, we first did a series of behavioural experiments to determine the acoustic parameters of probe tones. These control experiments were designed to choose probe-tone parameters so that the probe tones themselves did not override the behavioural context effect, while still evoking a reliable MEG response. We systematically varied the duration of probe tones, their presentation rate and the duration of the probe tone sequence (Supplementary Fig. 2). Based on the behavioural results, we selected sequences of 35-ms-long probe tones, presented every 250 ms (presentation rate of 4 Hz), with an overall sequence duration of 2 s. As a further control, we analysed the behavioural data collected during the main MEG experiment. Behavioural context effects observed during the MEG experiment were of similar magnitude as for previous experiments without the probe sequence, given the number of context tones and the time gap between context and test (Fig. 3e). There was an effect of the context sequence on *P*(Bias), as confidence intervals did not overlap with *P*(Bias)=0.5. When the same data were reanalysed using the probe tone frequency region to predict the bias, instead of the context frequency region, *P*(Bias) was only weakly above the 0.5 chance value (confidence interval overlapped with *P*(Bias)=0.5). These controls suggest that the probe tones did not interfere in any meaningful way with the context effect imposed by the context sequence.

The averaged MEG signals across participants showed time-locked responses to tones of the context, probe and test tones (Fig. 3b). During the probe sequence, this resulted in a periodic response at the probe tone presentation rate of 4 Hz. To quantify this response at 4 Hz for each participant, the sensor signals over the first 500 ms of the duration of the probe sequences were extracted from each individual trial and concatenated across trials. The first 500 ms were chosen as this is where the MEG response to individual probe tones was clearest, presumably because of response adaptation due to probe tones themselves (Fig. 3b), but analyses over longer durations gave similar outcomes. A discrete Fourier transform was then performed on the concatenated data. This produced a sharp peak at 4 Hz, as expected (Fig. 3c). According to the strength of this response at 4 Hz, 50 channels with the strongest power were chosen (Supplementary Methods, MEG Data analysis). The topography of this MEG response to the probe sequence (Fig. 3d) was consistent with typical responses for stimuli containing periodic amplitude modulation, suggesting a likely origin within sensory cortex^52^.

We aimed to characterize how neural responses to the probe tones were related to perceptual biases, on a trial-by-trial basis, and for identical stimulus configurations. To do so, trials were sorted according to whether the probe tones were presented to the same frequency region as the context (Context+ trials), or to the opposite frequency region (Context− trials). In each case, trials were further sorted according to whether the participant reported the expected pitch-shift direction induced by the context (Bias+), or whether the participant reported the opposite direction (Bias−). We finally contrasted the probe responses across the Bias+ and Bias− condition, which represent different perceptual outcomes for the same stimulus configuration. To account for intersubject variability in the absolute value of the MEG responses with the simplest normalization procedure, we computed a relative index summarizing the effect of context on the probe sequence. The probe response ratio (PRR) was defined as the 4-Hz probe response for Context+ trials, divided by the 4-Hz probe response for Context− trials. A PRR value lower than one would indicate a reduced responsivity in the frequency region of the context after presentation of the context, while a value above one would indicate enhanced responsivity. No effect of context would correspond to a PRR of 1.

Results are shown in Fig. 3f. In the Bias+ trials, corresponding to participants reporting the pitch shift expected from the context, the PRR was lower than one. In the Bias− trials, corresponding to listeners reporting a pitch shift opposite to what was expected from the context, the PRR was greater than one. This difference in PRR between Bias+ and Bias− trials was robust, as confirmed by a two-tailed paired Wilcoxon's signed-rank test (*W*=1, *P*<0.008, *N*=9).

This demonstrates an MEG correlate of the behavioural bias. For identical stimulus configurations, the PRR was reversed depending on the behavioural outcome. More specifically, the behavioural pitch shift encompassed the frequency region with relatively less MEG responsivity. Our design, intended to contrast identical stimulus configurations but different behavioural outcomes, only provides a relative measure of the response to the probe sequence, so it is not possible to discuss the effect of context in absolute terms. Previous studies using repeated pure tones have shown both suppression or enhancement of MEG responses following acoustic context^53^,^54^. Furthermore, relating MEG response magnitude to neural mechanisms such as adaptation is indirect, as computational modelling studies show that short-term synaptic adaptation over different timescales may result in either suppression or enhancement of MEG responses^55^. Nevertheless, the relative measure is sufficient to show that a behavioural bias is systematically associated to a reduction in MEG responsivity following the presentation of the context, all stimulus parameters being equal. More invasive recording techniques will be needed to clarify the link between this observation and mechanisms such as neural adaptation or enhancement^50^.

### A probabilistic model of temporal binding

What function might be served by the kind of contextual processing revealed by the behavioural biasing effects? Many otherwise surprising perceptual phenomena may arise from computational principles that reflect statistical properties of the natural world^56^,^57^. We constructed and simulated an inferential model to ask whether the same might be true of the context effects documented here.

The feature that listeners reported in our experiments—the direction of pitch shifts—necessarily depends on the comparison of sounds over time. Because the context effects were also observed for random-spectra stimuli (Fig. 2f), which do not produce a unitary pitch percept, it is likely that listeners reported frequency shifts between successive frequency components. The core idea of our model was that prior context may inform which successive frequency components were bound together, and therefore, which successive components were compared to estimate perceptual features such as pitch shift. The way prior context informed temporal binding was by simply assuming some degree of spectrotemporal continuity in the acoustics of sound sources: current frequency components are likely to be followed by future components at the same or proximal frequencies, because of persistence in the characteristics of sound sources.

**Figure 4:**
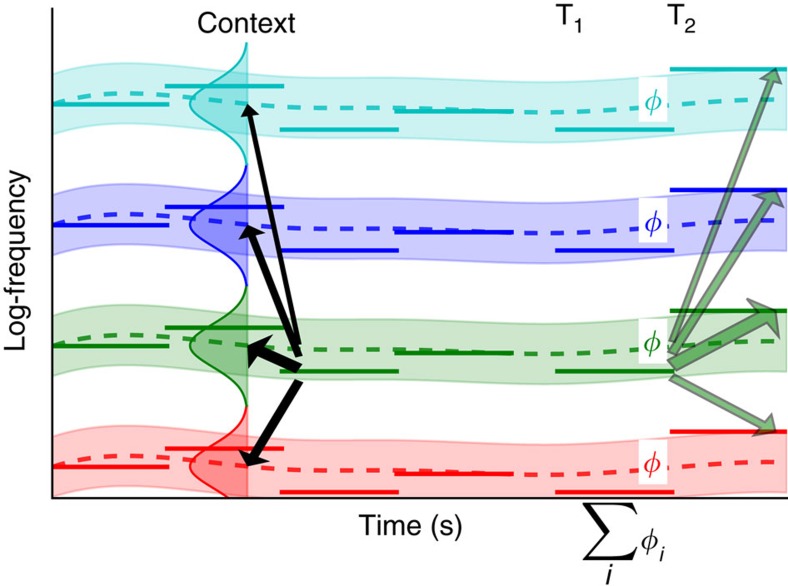
Probabilistic model. A track (colour coded) is instantiated for each of the component of the first context tone. Track means (dashed lines) are updated after each attribution for subsequent context tones. The coloured patch indicates the standard deviation of the underlying generative process. An attribution step is illustrated for the third tone of the context sequence (black arrows); arrow weights indicate the probability of attribution for each track. Using the same procedure, the final test tones components are attributed. Perceptual features (here, pitch shifts *φ*_*i*_) are finally computed within tracks. Green arrows represent all possible bindings from one component tone of T_1_ to component tones of T_2_; arrows thickness represents the likelihood that this binding was generated by the green track. The most likely binding correspond to a pitch shift encompassing the context-tone components, consistent with the perceptual bias.

This hypothesized process was implemented as inference within a probabilistic generative framework (Fig. 4). The model took as input a set of frequencies at different times, and assigned each observed frequency to what we termed a ‘track'. In the generative framework, the log frequencies of individual components were taken to be normally distributed with variance 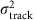 around the centre frequency associated with a track. The centre frequency of each track could evolve slowly through time. We also assumed that the internal representation of the frequency of each component was independently corrupted by sensory noise, with variance 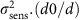 inversely proportional to the duration *d* of the tone, with *d*0 an arbitrary constant. The two variances, 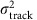 and 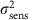, were the two free parameters of the model.

Responses to experimental stimuli were simulated by inferring the evolution of the model tracks, starting from the first context tone and ending with the final test tone. Tracks were initialized for each component of the first context tone, with a normal posterior belief about each centre frequency, the mean of which was set to the (noise-corrupted) component frequency and the variance of which was set to 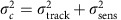. Inference for each subsequent tone then followed two steps: (1) the noisy component tones were probabilistically assigned to the tracks, in proportion to their probabilities of generation from them; (2) beliefs about the track centre frequencies were updated, according to the evidence provided by the assigned components weighted by their probabilities of assignment. Formally, this approach corresponded to mean-field filtering in a factorial hidden Markov model^58^. These filtering steps were iterated for each tone in the stimulus sequence. To compare the model with behavioural data, we finally predicted the perceived pitch shift between test tones by summing the shifts between every possible combination of test tones components, weighted by the inferred probability that the combination originated within the same track. The predicted pitch shifts thus reflected track assignments carried over from the first tone of the context sequence, and favoured pitch shifts within tracks (Fig. 4).

We fit the parameters 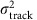 and 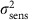 once for each listener, retaining the same values across all the different experiments in which he or she participated. A broad range of parameters provided an excellent fit to the data (Supplementary Fig. 3). The resulting model predictions are shown superimposed on the behavioural data in Figures 1 and 2. In most cases the confidence intervals between behaviour and model overlap. The model thus provides a single interpretative framework for most of the behavioural data, which we can now detail.

Without context, tracks were initialized at the components of the first test tone T_1_, and the highest probability was for each track to bind the component in the following tone T_2_ that was closest in log frequency, hence favouring the pitch shift over the smallest frequency distance between T_1_ and T_2_ (Fig. 1b). In the ambiguous case corresponding to an interval of 6 semitones (st), each component of T_2_ became equidistant on average from two tracks originating from T_1_; the symmetry was broken randomly by the simulated sensory noise, and so either shift direction was favoured equally often (Fig. 1b). Introducing a context tone made it more likely that the tracks initiated by the context would capture their neighbouring T_1_ and T_2_ components, and thus favour a pitch shift encompassing the context frequency region. The predicted bias was strongest when the context components, and thus prior track centres, fell halfway between the components of T_1_ and T_2_ as then the probability that the sensory noise would disrupt the context-induced tracks was smallest (Fig. 1d). Adding context tones increased the confidence in the track mean value (and their influence was more likely to average around the half-way point), thus increasing confidence in the assignment of T_1_ and T_2_ tones, consistent with the build-up of the effect with the number of context tones (Fig. 1f). The decrease in assumed sensory noise associated with longer tones was finally consistent with the effect of tone duration on context (Fig. 2b).

Using a single pair of parameters per subject, we achieved excellent quantitative agreement across the range of basic experiments. To maintain focus on the core mechanism, we did not attempt to model the data for the gap duration and random spectrum experiments, which would have required extensions to the model: the introduction of time-dependent corruption of sensory information for the gap data, and adaptive estimation of the variance of the generative process to handle arbitrary spectra. The success of the simple version of the model nevertheless supports the hypothesis that the context effect was based on temporal binding processes, enforcing statistical constraints of spectrotemporal continuity. Models with similar structure have been suggested before to account for auditory scene analysis^59^,^60^. The present framework is qualitatively different in what it aims to represent: the tracks identified by the model do not need to correspond to perceptually separated streams, which can be attended to at will^61^. Instead, many parallel tracks may be formed, implicitly, to guide the estimation of task-relevant perceptual features.

## Discussion

We have reported behavioural data showing that prior context can have a profound influence on a simple auditory judgement of pitch shift. Perceptual decisions could be fully swayed one way or another depending on prior context, for physically identical sounds and for pitch-shift values far from threshold. The existence region of the context effect, for timescales and spectral scales, was shown to encompass the prevalent statistics of natural auditory scenes. Brain imaging using MEG showed that the underlying neural trace manifested itself as a relative reduction in responsivity following the presentation of the context. Finally, a probabilistic model provided a functional interpretation for the context effects, in the form of temporal binding under the constraint of spectrotemporal continuity.

Our functional interpretation of the context effect is cast in terms of binding, and not auditory streaming, in spite of the apparent resemblance between the two notions^62^,^63^. This is because the behavioural effect was observed in conditions not usually associated with subjective streaming. An auditory stream takes some time to build up^64^, and it breaks apart for long pauses^65^ or large frequency discontinuities^66^. In contrast, we observed contextual effects in very short sequences, after very long silent intervals, and in the perception of very large frequency shifts. The current results thus reveal a form of binding processes outside of the parameter range of subjective streaming. This generalization is needed when one considers the situation faced by auditory perception. Even in the simplest case of a single auditory source, frequency components will be encoded by independent neural populations at the peripheral level. Because sound production is by nature dynamic, there will also be temporal gaps between those components. This introduces an inherent ambiguity: which component should be compared to which to estimate perceptual features? We suggest that the auditory system keeps track of the parallel evolution of frequency components in a way that maximizes continuity. This idea has strong similarities with what has been termed ‘serial dependency' in vision^8^, and is motivated by the same *a priori* continuity constraints on object persistence in the real world^67^.

The MEG findings showed the involvement of a neural trace within sensory regions, which manifested itself through a reduction in responsivity of the MEG response. Even though the link between MEG and neural responses is indirect^55^, a decrease in neural responses due to some form of adaptation seems a likely candidate for the underlying mechanism. It may seem paradoxical that a reduction in neural response should be associated with an enhanced probability to hear a perceptual feature. However, the perceptual bias we observed was not the subjective enhancement of the feature presented during context, which would perhaps be expected to be correlated to an increase in activity of the relevant neural population. Rather, the effect of the context was to guide temporal binding, and there was no *a priori* reason to predict whether the neural trace of the context should be of increase or decrease in neural activity. Suppressive traces have been found in short-term memory tasks with pure tone frequencies using functional magnetic resonance imaging^51^, and in neural responses with paradigms such as stimulus-specific adaptation^68^. Moreover, our generative model suggests that the context effect is linked to predictive processes, as temporal binding occurs between tones that best predict one another. Interestingly, in repetition-suppression paradigms, the response of the repeated tone is more suppressed for expected repeats compared with unexpected repeats^53^. Thus, memory traces, expectations and predictions may be correlated to reduced MEG responses.

Taken together, the behavioural, neural and computational evidence presented here suggest that each and every sound we hear imprints a predictive trace about the sounds that will follow. These traces are already expressed within sensory regions, perhaps mediated by adaptation over different timescales^47^,^69^. Crucially, such traces then modulate temporal binding and, as a direct consequence, the ongoing estimation of even the most basic of auditory features.

## Methods

### Experiment 1

*Participants.* Participants were 11 self-reported normal-hearing listeners (age: M=23.93 years, s.e.m.=0.17) who passed a screening procedure described in Supplementary Methods This experiment corresponds to Fig. 1a,b.

*Stimuli.* Shepard tones were chords made of a pure tone at base frequency, *F*_b_, superimposed with up to eight other octave-related pure tones and a fixed Gaussian amplitude envelope (central frequency=960 Hz, s.d.=1 octave). Each trial contained two tones, T_1_ and T_2_. To generate T_1_, a base frequency, *F*_b_, was randomly and uniformly drawn from 60 to 120 Hz, then the *F*_b_ for T_2_ was selected relative to T_1_, depending on the target interval between T_1_ and T_2_. The duration of each tone was 125 ms. The intertone interval (ITI) between T_1_ and T_2_ was 125 ms. Three tone complexes, which were similar to Shepard tones but with a half-octave spacing between components, were presented between trials to prevent the carryover of context effects.

*Procedure.* Ethical approval was provided by the CERES IRB No. 20142500001072 (Université Paris Descartes, France) for all behavioural experiments. Listeners read and signed a consent form before data collection. The task of the listener was to indicate whether T_1_ or T_2_ was higher in pitch. The T_1_–T_2_ interval was varied between 1 and 11 st. The experiment was divided into two blocks, with 220 trials per block and trials in randomized order. Responses were self-paced, and the delay between the listener's response and the next trial was set to 250 ms.

*Apparatus.* Listeners were tested individually in a double-walled sound-treated booth (Industrial Acoustics Company). Sound was delivered through an RME Fireface 800 sound card and 16-bit digital-to-analogue converter, at a 44.1 kHz sample rate. Stimuli were presented diotically through Sennheiser HD 600 headphones. Sound level was calibrated with a Bruel & Kjaer (2250) sound level meter and a Bruel & Kjaer ear simulator (4153). Stimuli in all experiments were presented diotically at 65 dB SPL (A-weighted).

### Experiment 2

*Participants.* Listeners were the same as in Experiment 1. This experiment corresponds to Fig. 1c–f.

*Stimuli.* Test stimuli were generated as in Experiment 1. The T_1_–T_2_ interval was fixed at 6 st. To generate the context, two half-octave wide frequency regions were defined relative to the components of T_1_ and T_2_: half an octave above the *F*_b_ of T_1_, or half an octave below the *F*_b_ of T_1_. In a given trial, the context consisted of Shepard tones selected from one of the two regions, with an *F*_b_ drawn randomly from a uniform distribution. Each context tone lasted 125 ms with an ITI of 125 ms between context tones and an ITI of 500 ms between the context and test. Sequences of 10 intertrial tones, generated as in Experiment 1, were presented between trials.

*Procedure.* The number of context tones was varied between 0 and 10 tones. The experiment was divided into two blocks, with 220 trials per block. Apparatus and other experimental details are as in Experiment 1.

### Experiment 3

*Participants.* One hundred listeners, unscreened and self-recruited through a research network (RISC, http: //www.risc.cnrs.fr) completed the experiment online. This experiment corresponds to Supplementary Fig. 1.

*Stimuli.* Main trials (Context-T_1_–T_2_) were generated as in Experiment 2, with 10 context tones. Test trials were also included, containing two Shepard tones, T_1_ and T_2_, with an interval of either 1 st (‘up') or 11 st (‘down') to evaluate the performance of listeners on pitch shift judgements. Catch trials finally evaluated the engagement of listeners, using an identical structure to the standard Context-T_1_–T_2_ trials but with sounds containing the first 100 harmonics of a fundamental frequency *F*0 and thus producing unambiguous up or down pitch motions.

*Procedure.* The experiment consisted of a block of 22 test trials followed by a second block containing 44 main trials and 6 catch trials. Trial order was randomized within blocks.

### Experiment 4

*Participants.* Ten self-reported normal-hearing listeners participated (age: M=24.40 years, s.e.m.=0.24) This experiment corresponds to Fig. 2a,b.

*Stimuli.* Stimuli were generated as in previous experiments. Each trial followed the format Context T_1_–T_2_. The context consisted of one Shepard tone with an interval of 3 st or −3 st with respect to T_1_. The duration of T_1_ and T_2_ was 125 ms with no ITI between T_1_ and T_2_ or between the context and T_1_. The duration of the context tone was the experimental variable. It could take any of the following values: 0, 5, 10, 20, 40, 80, 160 and 320 ms. Intertrial sequences of 10 tones were presented.

*Procedure.* There were 40 repetitions per context-tone duration condition, leading to a total of 320 trials presented in random order. Other experimental details as in Experiment 1.

### Experiment 5

*Participants.* Ten self-reported normal-hearing listeners (age: M=26.33 years, s.e.m.=0.49) This experiment corresponds to Fig. 2c,d.

*Stimuli.* Stimuli were generated as in Experiment 2, following the format Context-T_1_–T_2_. The number of context tones was fixed at five tones. The ITI between the context and T_1_ was the experimental variable and could take any of the following values: 0.5, 1, 2, 4, 8, 16, 32 and 64 s.

*Procedure.* There were 20 repetitions per Context-T_1_ ITI condition, leading to a total of 160 trials presented in random order. Other experimental details were as in Experiment 1.

### Experiment 6

*Participants.* Ten self-reported normal-hearing listeners participated (age: M=26.85 years, s.e.m.=1.10) This experiment corresponds to Fig. 2e,f.

*Stimuli.* Stimuli were inharmonic complexes with randomly spaced components of equal amplitude, consisting of *N* components. Each trial followed the format Context-T_1_–T_2_. In each trial, T_1_ consisted of a reference chord with randomly drawn frequencies between 30 Hz and half the sampling frequency (22,050 Hz). T_2_ and Context were generated with respect to T_1_, by shifting components relative to the spacing between neighbouring components of T_1_. Additional information is given in the Supplementary Methods.The number of components within each chord, *N*, was an experimental parameter. All chords had a duration of 125 ms.

*Procedure.* In each trial, participants indicated whether T_1_ or T_2_ was ‘higher in pitch'. In a no-context condition, each trial consisted of a test pair, T_1_–T_2_, presented without any preceding context. In the context condition, the test pair, T_1_–T_2_, was preceded by a sequence consisting of five context tones. The number of components, *N*, could take the values of 3, 5, 10, 20, 40 or 80. Trial order was randomized and there were two blocks each with 20 repetitions per condition, resulting in a total of 480 trials. Other experimental details were as in Experiment 1.

### MEG experiment

*Participants.* Nine self-reported normal-hearing listeners (age: M=24, s.e.m.=1.2) participated This experiment corresponds to Fig. 3.

*Stimuli.* Stimuli were generated in the same manner as in previous experiments, except that a flat amplitude envelope was used. Each trial followed the format Context-T_1_–T_2_. The context consisted of eight Shepard tones, each with a tone duration of 125 ms. ITIs were set to 125 ms. A probe sequence was inserted between the context and T_1_–T_2_, to measure the adaptive trace left by the context. The probe sequence consisted of eight Shepard tones each with a duration of 35 ms and with a 215-ms ITI between probe tones. The probe tones were presented to the same frequency region as the context (Context+), or to the frequency region where no context tones were presented (Context−).

*Procedure.* Experimental procedures were approved by the University of Maryland Institutional Review Board. Listeners read and signed a consent form before data collection. The task was to indicate whether T_1_ or T_2_ was higher in pitch. Listeners performed the task while neural activity was recorded using MEG. The frequency region of the context and the frequency region of the probe sequence relative to the context were experimental variables. Conditions were repeated 30 times in total, leading to 360 trials in total, and trial order was randomized. The intertrial interval was varied randomly between 800 and 1,000 ms. The sound level was set to 70 dB SPL.

*Apparatus.* Sound was delivered to participants using a M-Audio Audiophile 2,496 sound card and 50-V sound tubing (E-A-RTONE 3A; Etymotic Research), attached to E-A-RLINK foam plugs inserted into the ear canal. MEG recordings were performed in a magnetically shielded room (Yokogawa Electric Corporation) using a 160-channel whole-head system (Kanazawa Institute of Technology, Kanazawa, Japan), and with a sampling rate of 1 kHz. Further details are given in the Supplementary Methods.

### Probabilistic model

We constructed a probabilistic model of the perception of frequency shifts between tone complexes. The model took as input a noisy sequence of component tone frequencies and used a factorial hidden Markov model, to cluster these components into ‘tracks' on the basis of their spectrotemporal continuity, in three stages: initialization, tracking and construction of the shift percept The model is illustrated in Fig. 4, and its predictions in panels of Fig. 1 and Fig. 2.

*Initialization.* A chord initiates one track per component tone, but maintains uncertainty in the form of a Gaussian distribution about the central frequency of the track. The variance is the sum of the variance of acoustic frequencies associated with any one track (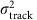) and the variance of the sensory noise that corrupts the sensed frequency (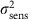). These two free parameters of the model were estimated from the behavioural data. For each sensed tone we computed sensory noise reduction with tone duration 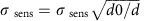, where *d*0 is a reference tone duration (150 ms) and *d* is the sensed tone duration.

*Tracking.* As new chords are input into the model, the component tones are attributed to the different tracks, and the belief about the mean of each track is then updated to incorporate the new observed frequencies (Fig. 4). In a ‘soft' process, each tone is partly attributed to all tracks. Tracks closer in frequency to a given component tone assume greater ‘responsibility' for it. Responsibility is the probability under the model that a given tone with frequency *g*_*j*_ might have arisen from a track *i*. We introduce an attribution label *η*_*j*_ which is the (unknowable) identity of the track which actually generated tone *j*. Then, the responsibility is the probability that *η*_*j*_=*j*. That is, if ongoing beliefs concerning the mean frequency are defined by {{*μ*_1_, *σ*_1_},…,{*μ*_*k*_, *σ*_*k*_}} and the chord *c* is presented, we compute for each tone *j* and each track *i*, the responsibility 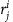 as follows:


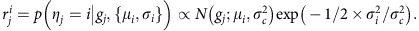


Once attribution has taken place, the mean frequency of each track is updated with the frequencies of all tones, weighted by the responsibilities. We update the beliefs about the ongoing tracks as follows: for each track *i*, we compute the effective number of tones attributed to that track, 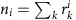 and the weighted mean frequency of the tones attributed to that track: 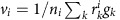. Mean and variance of the belief about track *i* are updated as follows: 
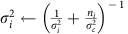
, 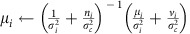. This process of attribution and updating is repeated for all remaining stimuli in the trial.

*Shift percept construction.* Finally, we modelled the behavioural response of the listener when judging the direction of pitch shift between a pair of consecutive chords. Frequency shifts were computed locally within each track, and these local shifts were then combined across tracks to build a global percept of pitch change. The local frequency shift within a track was taken to be the sum over all possible oriented shifts between pairs of consecutive tones in the two chords, weighted by how likely they were to both belong to that track. For track *i*, 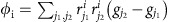. The global shift percept was then simply the sum of the track-local shifts: 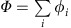. A binary percept was constructed by thresholding *Ф* at 0. When *Ф* is positive, a rising pattern is predicted, and when Ф is negative, a falling pattern is predicted.

### Data availability

The datasets generated and analysed during the current study are available from the corresponding author on reasonable request.

## Additional information

**How to cite this article:** Chambers, C. *et al*. Prior context in audition informs binding and shapes simple features. *Nat. Commun.* **8**, 15027 doi: 10.1038/ncomms15027 (2017).

**Publisher's note:** Springer Nature remains neutral with regard to jurisdictional claims in published maps and institutional affiliations.

## Supplementary Material

Supplementary Information Supplementary Figures, Supplementary Tables, Supplementary Methods and Supplementary References

Supplementary sound file Audio S1 Illustration of a 10-tones context sequence,

Supplementary sound file Audio S2 Same as Audio S1, but with context tones now
