# Supplementary material for: Prior context in audition informs binding and shapes simple features
Source: Nat Commun. 2017 Apr 20;8:15027. doi: 10.1038/ncomms15027 (PMC5411480; doi:10.1038/ncomms15027)
Supplement: Supplementary Information — Supplementary Figures, Supplementary Tables, Supplementary Methods and Supplementary References [file ncomms15027-s1.pdf]

Supplementary Data

Supplementary Table 1. Supplementary analyses for Fig. 1d presenting mean $P(T_1H)$ and bootstrapped Bonferroni-corrected (1 - α) × 100% confidence intervals for different context intervals.

Context interval (lowest, highest interval)	*Mean (CI)*
-6, -5	.63 (.35, .82)
-5, -4	.69 (.44, .88)
-4, -3	.78 (.56, .92)*
-3, -2	.85 (.44, 1)
-2, -1	.78 (.42, .1)
-1, 0	.45 (.17, .77)
0, 1	.34 (.10, .69)
1, 2	.28 (.03, .64)
2, 3	.12 (.01, .29)*
3, 4	.09 (.00, .25)*
4, 5	.17 (.03, .40)*
5, 6	.34 (.11, .67)

* CI does not overlap with .5

Supplementary Table 2. Supplementary analyses for Fig. 1f presenting mean P(Bias) and bootstrapped Bonferroni-corrected (1 - α) × 100% confidence intervals for different numbers of context of tones.

# Context tones	*Mean (CI)*
1	.73 (.61, .83)*
2	.84 (.71, .93)*
3	.91 (.80, .96)*
4	.92 (.85, .95)*
5	.94 (.87, .97)*
6	.94 (.88, .97)*
7	.95 (.90, .98)*
8	.95 (.91, .99)*
9	.96 (.93, .98)*
10	.95 (.91, .98)*

* CI does not overlap with .5

Supplementary Table 3. Supplementary analyses for Fig. 2b presenting mean P(Bias) and bootstrapped Bonferroni-corrected (1 - α) × 100% confidence intervals for different context tone durations.

Duration of C (ms)	*Mean (CI)*
5	.53 (.46, .64)
10	.49 (.44, .53)
20	.60 (.56, .66)*
40	.76 (.56, .90)*
80	.81 (.64, .93)*
160	.90 (.80, .98)*
320	.89 (.75, .98)*

* CI does not overlap with .5

Supplementary Table 4. Supplementary analyses for Fig. 2d presenting mean P(Bias) and bootstrapped Bonferroni-corrected (1 - α) × 100% confidence intervals for different gap durations between context and test.

C- T_1 delay (s)	*Mean (CI)*
0.5	.94 (.86, .98)*
1	.93 (.89, .98)*
2	.92 (.87, .97)*
4	.86 (.81, .93)*
8	.79 (.68, .88)*
16	.78 (.68, .87)*
32	.73 (.63, .84)*
64	.64 (.49, .79)

* CI does not overlap with .5

Supplementary Table 5. Supplementary analyses for Fig. 2f presenting mean P(Bias) and bootstrapped Bonferroni-corrected (1 - α) × 100% confidence intervals for different component densities.

# Components/octave	*Mean (CI)*
0.32	.91 (.84, .95)*
0.53	.96 (.93, .98)*
1.05	.98 (.94, 1.00)*
2.10	.96 (.84, 1.00)*
4.20	. 87 (.74, .96)*
8.40	.62 (.51, .73)*

* CI does not overlap with .5

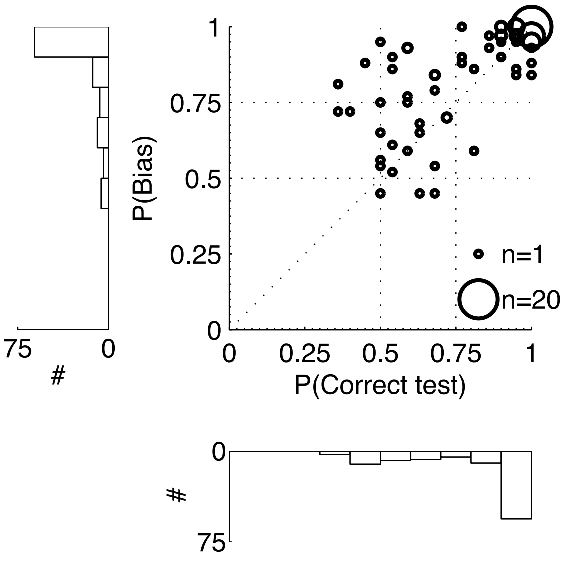

Supplementary Figure 1. Related to online experiment with large sample size.

Results for the online Experiment 3, for N=100 listeners. The P(Bias) is plotted a function of P(Correct), the proportion correct on test trials consisting of unambiguous pitch changes of 1 semitone. The size of the marker represents the number of listeners. No context effect corresponds to P(Bias)=0.5; chance for pitch direction identification corresponds to P(Correct)=0.5. Dotted lines mark these values, as well as the 0.75 proportion in each case and the identity diagonal. Count histograms are also displayed for P(Bias) and P(Correct).

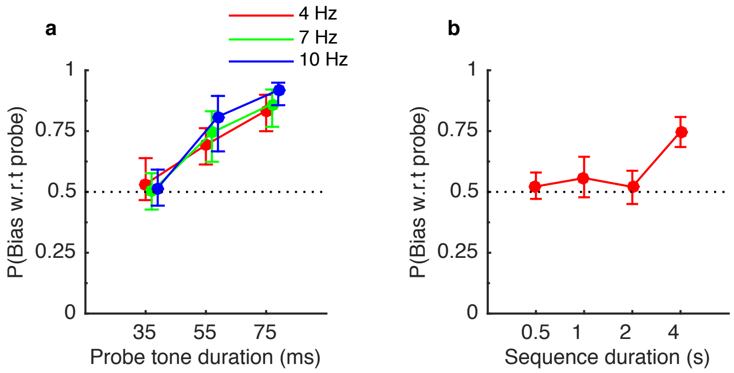

Supplementary Figure 2. Psychophysics relating to neural correlates of bias.

A pilot experiment was run to determine the acoustical parameters of the probe tones that would interfere minimally with the bias due to the context sequence. Ten participants with self-reported normal hearing were tested (6 females, mean age $M = 26$ y, $SD = 2.8$) in a sound-attenuated booth (J.W. Manny Inc., Eckel Sound Rooms). Sounds were played using an Intel HD Audio sound card with 44.1 kHz output sample rate and 24-bit sample depth, delivered diotically using Sony MDR-V700 headphones. Stimuli were as in the main MEG experiment, except for the parameter varied for the pilot experiment. In all panels error bars represent the 95% confidence intervals of the mean across participants. (**a**) We first varied the duration of each probe tone and the probe tone presentation rates. Tone duration was the main determinant of perceptual bias, with a main effect of tone duration ($H(2)=16.2$, $p<.0005$, $N=10$, Fig. 3a). Shorter tones produced smaller biases, with no measurable perturbing effect on the perceptual bias for probe tone durations of 35 ms. Presentation rate had no effect ($H(2)=1.8$, $p>.05$, $N=10$). We selected the 4-Hz presentation rate and 35-ms duration and performed a second control experiment, where we varied the duration of probe sequences. (**b**) Sequence duration had an effect on behavioral bias ($H(3)=14.89$, $p<.005$, $N=10$, Fig. 3b). A decrease in the context effect caused by the Probe sequence was only visible for the longest, 4-s sequences. We thus selected a duration of 2 s for the MEG experiment.

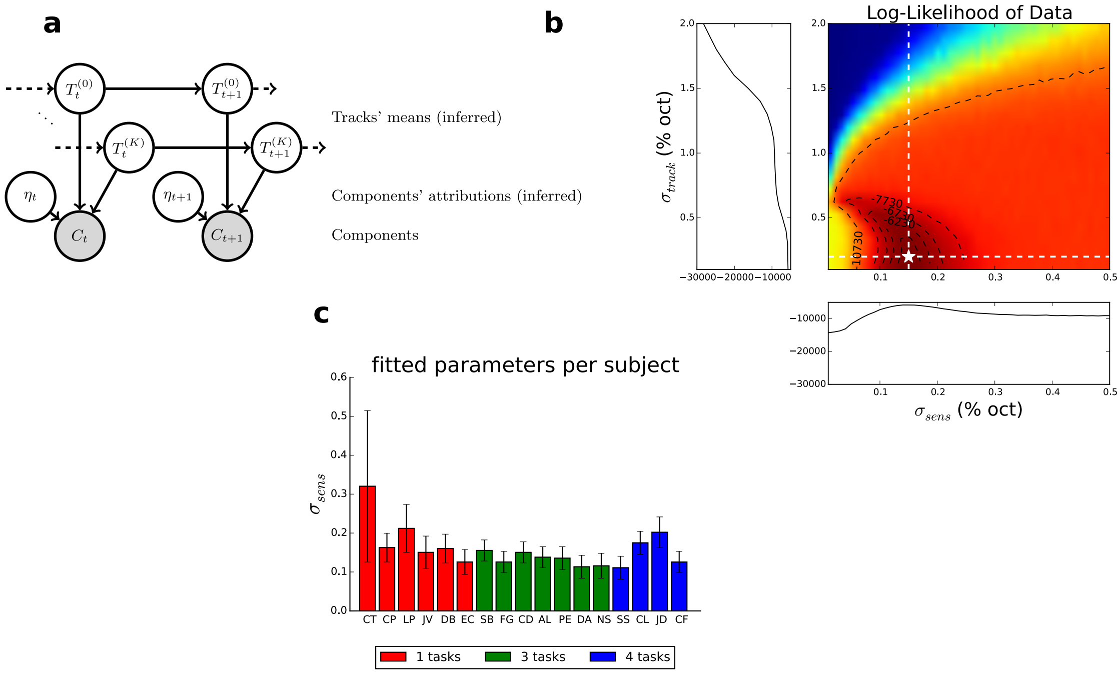

Supplementary Figure 3. Related to Fig. 4. Probabilistic model.

(a) Graphical model of the generative process underlying the formation of tracks by the probabilistic model. Arrows denote statistical conditional dependencies. Horizontal arrows describe the temporal continuity of tracks (T) that are hypothetized to generate the spectro-temporal components (C) constituting the scene. Each component belongs to a single track whose identity (η) is inferred. **(b)** Log-likelihood of the psychophysical data as a function of model parameters, summed across participants. A broad range of parameters provides a good fit to the data. Dashed lines in black represent iso-value curves of the log-likelihood. The side panels represent the log-likelihood along the two white dashed lines in the main panel. In the left panel, the parameter σ_{sens} is fixed to the best-fitting value. For the parameter σ_{track}, values up to 0.5 provided a similar fit. Therefore, σ_{track} was set to an arbitrary value in this range (0.16) in the bottom panel. **(c)** A bar chart displays best-fitting parameter σ_{sens} for individual subjects. The error bars display the narrowest 95% confidence interval around the mode of the normalized likelihood, which provide an estimate of the estimation uncertainty.

Supplementary Methods

The Supplementary Methods present an expanded version of the main Methods section, containing the original description together with additional details.

Experiment 1: Shepard tone pairs without context (Fig. 1a, b)

Participants

Sixteen self-reported normal-hearing listeners (12 female, 4 male) participated with a mean age of 23.93 years (SEM=0.17). Five participants were excluded as they did not pass a screening test. This test involved reporting the direction of 1-semitone pitch shifts between pure tones and Shepard tones. Individual variability has been reported before on such a task[1]. Performance of 80% or above was required to take part in the main experiment[2]. No data was collected for listeners who failed the screening test. Note that in the subsequent Experiment 3, no screening was applied for a larger group of listeners.

Stimuli

Shepard tones

Shepard tones were generated as in a previous study[2]. Briefly, they were chords made of a pure tone at base frequency, $F_{b,}$ superimposed with up to 8 other pure tones all with octave relationships to each others ($F_{b,}$ $2F_{b,}$ $4F_{b,}$ $8F_b$ etc.). A fixed amplitude envelope was applied (Gaussian shape on a log-amplitude scale, centered at 960 Hz, with +/1 SD deviation corresponding to 1 octave and 6 dB down-points of 424.58 Hz and 2170.62 Hz).

Shepard tone pairs

Each trial contained two tones, T_1 followed by T_2. A base frequency, $F_{b,}$ was randomly drawn (uniform distribution) from 60 to 120 Hz for T_1. The F_b of T_2 was selected relative to T_1, depending on the target interval between T_1 and T_2. The duration of each tone was 125 ms. The inter-tone interval (*ITI*) between T_1 and T_2 was 125 ms. All other details as in a previous study[2].

Inter-trial sequences

We aimed at preventing any potential carry-over of context effects across trials by presenting "resetting" tone sequences between trials. Sequences of three tone-complexes were presented, with the same characteristics as Shepard tones but with a half-octave spacing between components. This ensured a balanced coverage of positive and negative hypothetical biases.

Procedure

Ethical approval was provided by the CERES IRB #20142500001072 (Université Paris Descartes, France) for all behavioral experiments. Listeners read and signed a consent form prior to data collection. The task of the listener was to indicate whether T_1 or T_2 was higher in pitch. The T_1-T_2 interval was varied between 1 and 11 semitones (st). One musical semitone is approximately a 6% change in frequency. There were 40 repetitions per condition, leading to a total of 440 trials. The experiment was divided into two blocks, with 220 trials per block. The order of trials was randomized. Responses were self-paced, and the delay between the listener's response and the next trial was set to 250 ms.

Apparatus

Listeners were tested individually in a double-walled sound-treated booth (Industrial Acoustics Company). Stimuli were generated using custom programs on a personal computer, using Matlab. They were delivered through a RME Fireface 800 sound card and 16-bit digital-to-analogue converter, at a 44.1 kHz sample-rate. Stimuli were presented diotically through Sennheiser HD 600 headphones. Sound level was calibrated with a

Bruel & Kjaer (2250) sound level meter and a Bruel & Kjaer ear simulator (4153). Stimuli in this experiment and all remaining experiments were presented at 65 dB SPL (A-weighted). Stimuli were presented diotically.

Experiment 2: Context sequence (Fig. 1c, d, e, f)

Participants

Listeners were the same as in Experiment 1.

Stimuli

Test stimuli

Test stimuli were generated in the same manner as in Experiment 1. The T_1-T_2 interval was fixed at 6 st for the duration of the experiment.

Context stimuli

The test was preceded by a sequence of contextual Shepard tones. In order to generate the context, two half-octave wide frequency regions were defined relative to the components of T_1 and T_2: half an octave above the F_b of T_1, or half an octave below the F_b of T_1. In a given trial, the context consisted of Shepard tones selected from only one of the two possible frequency regions, with an F_b drawn randomly from a uniform distribution. Each context tone lasted 125 ms with an *ITI* of 125 ms between context tones. The *ITI* between the context and test was set to 500 ms.

Inter-trial sequences

Sequences of 10 tones were presented between trials, generated in the same manner as in Experiment 1.

Procedure

The number of context tones was the experimental variable. It could take any value between 0 and 10 tones. There were 40 repetitions number of context tones condition, leading to a total of 440 trials presented in random order. The experiment was divided into two blocks, with 220 trials per block. Apparatus and other experimental details are as in Experiment 1.

Experiment 3: Online experiment (Supp. Fig. S1)

Participants

Participants were recruited through the mailing list of a research network (RISC, www.risc.cnrs.fr). One hundred listeners completed the experiment, after which we stopped collecting data. No demographic data was collected for anonymity reasons.

Stimuli

Test trials

Test trials were included to evaluate the performance of listeners on non-ambiguous pitch shift judgments. Test trials contained only two Shepard tones, T_1 and T_2, with an interval of either 1 st ("up") or 11 st ("down").

Main trials

Context-test sequences were generated in the same manner as Experiment 2. The number of context tones was fixed at 10 tones.

Catch trials

Catch trials were included to evaluate the involvement of listeners in the online task. Harmonic complexes were generated, consisting of the first 100 harmonics of a fundamental frequency ($F0$). The spectral envelope was the same as that used for the Shepard tone stimuli. Sequences of harmonic complexes were used as non-ambiguous analogs of the Shepard context-test stimuli. Each trial had an identical structure to the standard Context-T_1-T_2

trials, except that variations in F_b were replaced by variations in $F0$, resulting in a clear up or down pitch motion. Results on these catch trials were always high (89% above 75%) so they did not lead to removing any full dataset from the analysis.

Procedure

Participants conducted the experiment online at a website provided by the RISC. Listeners first carried out a block of 22 trials where their ability to report pitch shifts would be evaluated (test trials). Listeners then completed a second block of 50 trials, where each trial consisted of a context-test sequence. On 44 of the trials, Shepard tone context-test stimuli were presented (main trials). The remaining 6 trials consisted of harmonic complexes context-test sequences (catch trials). Trial order was randomized within blocks. No feedback was provided at any stage.

<u>Experiment 4: Short context (Fig. 2a, b)</u>

Participants

Fifteen self-reported normal-hearing listeners (7 female, 8 male) with a mean age of 24.4 years (SEM = 0.24) participated in the experiment, of which five were excluded as they did not pass the screening test.

Stimuli

Stimuli were generated in the same manner as in previous experiments. Each trial followed the format Context-T_1-T_2, as in Experiment 2. *C* consisted of one Shepard tone with an interval of 3 st or -3 st with respect to T_1. The duration of T_1 and T_2 was 125 ms with no *ITI* between T_1 and T_2 or between the context and T_1. Inter-trial sequences of 10 tones were presented between trials.

Procedure

The duration of the context tone was the experimental variable. It could take any of the following values: 0, 5, 10, 20, 40, 80, 160 and 320 ms. Raised cosine onset and offset ramps were set to a 5-ms duration in all conditions, except when tone duration was 10 ms or lower, in which case ramp duration was reduced to 2.5 ms. There were 40 repetitions per context-tone duration condition, leading to a total of 320 trials presented in random order. Apparatus and other experimental details as in Experiment 1.

<u>Experiment 5: Long gap (Fig. 2c, d)</u>

Participants

Ten self-reported normal-hearing listeners (5 female, 5 male) with a mean age of 26.33 years (SEM = 0.49) participated in the experiment. None of the listeners were excluded based on their performance on the screening test.

Stimuli

Stimuli on each trial were generated in the same manner as Experiment 2, following the format Context-T_1-T_2.

Procedure

The *ITI* between the context and T_1 was the experimental variable. It could take any of the following values: 0.5, 1, 2, 4, 8, 16, 32, and 64 s. The number of context tones was fixed at 5 tones. There were 20 repetitions per Context-T_1 *ITI* condition, leading to a total of 160 trials presented in random order. Apparatus and other experimental details as in Experiment 1.

Experiment 6: Random spectra (Fig. 2e, f)

Participants

Fourteen self-reported normal-hearing listeners (6 female, 8 male) with a mean age of 26.85 years (SEM = 1.10) participated of which four were excluded as they did not pass the screening test.

Stimuli

Stimuli were inharmonic complexes with randomly-spaced components. Components were equal in amplitude. For a given complex tone, N components were generated between the lowest frequency *FL* (set at 30 Hz) and half the sampling frequency, *sf*/2 = 22050 Hz with equal amplitude. Each trial followed the format Context-T_1-T_2. In each trial, T_1 consisted of a reference chord with randomly drawn frequencies. T_2 and Context were generated with respect to T_1. The frequency of each component, *i*={0, 1, 2,..., N}, of T_1 was computed as:

$$\mathrm{f_i} = 2^{\mathrm{cs_i}}.\mathrm{FL}$$

where $cs_\mathrm{i} = k\frac{(i+x_\mathrm{i})}{N}$ and $k = \log_2\frac{sf}{2FL}$, with x_i representing independent, uniformly distributed random numbers between 0 and 1. In order to generate ambiguous shifts between consecutive tones, the frequencies of each new complex were shifted with respect to neighboring components of T_1. The frequency of each component of the context tone and T_2, *j*={0, 1, 2,... , *N*}, was generated with respect to the components of the reference chord in the following manner:

$$\mathrm{f_j} = 2^{\mathrm{cc_j}}.\mathrm{FL} \quad \text{where} \quad \mathrm{cc_j} = \mathrm{cs_j} + \mathrm{y}(\mathrm{cs_{j+1}} - \mathrm{cs_j})$$

y being a number between 0 and 1, specifying the relationship between the components of the reference and the chord being generated.

The factor *y* is similar to the interval in st of previous experiments. It was set to 0.5 for T_2 and during the context was randomly varied from 0 to 0.5 or 0.5 to 1. The number of components within each chord, *N*, was an experimental parameter. All chords had a duration of 125 ms, including 5-ms raised-cosine onset and offset ramps. The *ITI* between context tones was 312 ms. The *ITI* was 312 ms between the context sequence and the test pair. The *ITI* between test tones was 125 ms.

Procedure

In each trial, participants indicated whether T_1 or T_2 was "higher in pitch". In a no-context control condition, each trial consisted of a test pair, T_1-T_2, presented without any preceding context. In the main context condition, the test pair, T_1-T_2, was preceded by a sequence consisting of 5 context tones. The number of components, *N*, could take the values of 3, 5, 10, 20, 40, or 80. These values correspond to an average spacing of 0.32, 0.53, 1.05, 2.10, 4.20, and 8.40 components per octave, respectively. All conditions were interleaved randomly within a block. There were 40 repetitions for each condition, resulting in a total of 480 trials. Apparatus and other experimental details as in Experiment 1.

Analysis of behavioral data

Since the data did not meet the requirements for parametric tests due to heteroscedasticity (in data shown in Figure 2B and 2F) and lack of normality of residuals, we performed non-parametric Kruskal-Wallis tests on mean ranks to examine main effects for all experiments.

Permutation tests were used where we compared the H statistic computed from the original dataset to that obtained from 10,000 null datasets. Null datasets were generated by randomly exchanging condition labels within each participant. The reported p-value equals the proportion of cases where the null H statistic exceeded

the H statistic computed from the original dataset. In the main text, the H statistic from the original data set is reported with the p-value computed from the permutation test.

In order to compare the P(Bias) in individual conditions to the chance level of 0.5, bootstrapped confidence intervals were computed, which were then Bonferroni-corrected for the number of comparisons ((1 – 0.05/# comparisons) × 100%).

MEG experiment (Fig. 3)

Participants

5 female and 4 male self-reported normal-hearing listeners with a mean age of 24 years (SEM = 1.2) participated.

Stimuli

Stimuli were generated in the same manner as in previous experiments, except that a nominally flat spectral envelope was applied (all amplitudes of chord components were equal). Each trial followed the format Context-T_1-T_2, as in Experiment 2. A probe sequence was inserted between the context and T_1-T_2, in order to measure the adaptive trace left by the context during the MEG experiment. The context consisted of eight Shepard tones, each with a tone duration of 125 ms. *ITI*s were set to 125 ms. Context sequences were as in Experiment 2. The probe sequence consisted of eight Shepard tones each with a duration of 35 ms and with a 215-ms *ITI* between probe tones (presentation rate: 4 Hz). The probe sequence could be presented either at the center of the same frequency region as the context (Context+) where the recorded MEG response was expected to be influenced by the context sequence, or at the center of the frequency region where no context tones were presented (Context-) which served as a baseline condition. The short duration of the probe tones was intended to minimize their impact compared to the context sequence.

Procedure

The experimental procedures were approved by the University of Maryland Institutional Review Board. Listeners read and signed a consent form prior to data collection. Before MEG recordings, stimuli were optimized to maximize contextual effects for each listener, as the base frequency of the tritone Shepard tone pair may influence its degree of ambiguity[3]. Listeners adjusted the sound level to a comfortable level before starting the experiment. As for previous experiments, each trial was of the form Context-T_1-T_2 without the probe sequence and the task was to indicate whether T_1 or T_2 was higher in pitch. We selected the three base frequencies which maximized context effects for each individual, then only presented these during the MEG recordings that followed. The base frequency was varied between 440 Hz and 830.61 Hz, with intervals of 1 semitone between conditions, leading to 12 base frequency conditions (0 – 11 semitones, re: 440 Hz). There were 10 repetitions per condition, leading to a total of 120 trials.

During the second part of the experiment, listeners performed the same task while neural activity was recorded using MEG. Probe sequences were included between the context and T_1-T_2. The frequency region of the context and the frequency region of the probe sequence relative to the context were experimental variables. The base frequency could take on three values. Conditions were repeated 30 times in total, leading to 360 trials in total, and trial order was randomized. The inter-trial interval was varied randomly between 800 and 1000 ms. The sound level was set to 70 dB SPL.

Apparatus

Participants conducted the first portion of the experiment in a sound-attenuated booth (J.W. Manny Inc., Eckel Sound Rooms). Sound was delivered using an Intel HD Audio sound card with 44.1 kHz output sample rate and 24-bit sample depth. Sony MDR-V700 headphones were used. During the subsequent MEG recordings, sound was delivered to participants using a M-Audio Audiophile 2496 sound card and 50-V sound tubing (E-A-RTONE 3A; Etymotic Research), attached to E-A-RLINK foam plugs inserted into the ear canal. The entire acoustic delivery system was equalized to give an approximately flat transfer function from 40– 3,000 Hz. MEG recordings were performed in a magnetically shielded room (Yokogawa Electric Corporation) using a 160-channel whole-head system (Kanazawa Institute of Technology, Kanazawa, Japan), and with a sampling rate of 1 kHz. Detection coils were arranged in a uniform array on a helmet-shaped surface, with ~25 mm between the centers of two adjacent 15.5-mm-diameter coils. Sensors were configured as first-order axial gradiometers with a baseline of 50 mm; their field sensitivities were 5 fT/√Hz or better in the white noise region. A 200 Hz low-pass filter and a notch filter at 60 Hz were applied to the magnetic signal online. Three of the 160 channels were magnetometers separated from the others and used as reference channels to measure and cancel environmental noise. Five electromagnetic coils were used to measure each subject's head position inside the MEG machine. The head position was measured twice during the experiment, once before the experiment and once after the experiment to quantify the head movement.

Data analysis

To analyze MEG recordings, the first 500 ms of each 2-s probe sequence was extracted. Only the first 500-ms time window was selected to minimize adaptation effects from the probe sequence itself on the recorded MEG response (see Figure 3b), but similar results were obtained with longer windows. Neural responses from all trials were then concatenated to obtain an extended response with duration T = [500 ms × number of trials] for each time window and for each channel. Concatenated responses were represented in the frequency domain using a Discrete Fourier Transform (DFT), yielding a frequency spectrum from 0 to 500 Hz at a resolution of $1/T$ Hz. The complex magnetic field strength was obtained by using the product of the DFT and the sampling interval ($1/SR$). Power spectral densities were computed by squaring the complex magnetic field strength, normalized by the duration of the signal. We then calculated the square magnitude of the frequency component at 4 Hz, and averaged over the 50 channels with the strongest normalized responses for each participant.

Topography was analyzed using the method of Simon & Wang (2005). To obtain contour plots, the complex responses at 4 Hz were projected on lines of constant phase [4].

Neural recordings of all trials were sorted according to the frequency region of the probe sequence relative to the context (Context+ and Context-) and then further divided according to whether the listener's response was in accordance with the contextual bias (Bias+) or whether their response was in the opposite direction (Bias-), leading to four categories: Bias+Context+, Bias+Context-, Bias-Context+, and Bias-Context-.

In order to have a fair cross-conditional comparison of the results, an equal number of trials for each condition was analyzed. This addressed the dependence of the *SNR* on the number of trials, and the variation of the number of trials for each condition per participant (based on individual performance). Trial equalization was done by performing the analysis on the minimum number of trials available for conditions, and then bootstrap resampling was applied for those conditions with a higher number of trials and averaged over the results of bootstrapping to obtain the final neural response power. Across subjects, this gave an average number of trials

and standard deviation of 248 (56), 112 (56), 242 (53), 117 (53), for categories Bias+Context+, Bias+Context-, Bias-Context+, and Bias-Context-, respectively.

A Probe Response Ratio (*PRR*) was as the response at 4Hz in Context+ trials, divided by the 4-Hz probe in Context– trials, for each participant, and without any normalization. The *PRR* was computed separately for Bias+ and Bias– trials.

Probabilistic model (Fig. 4)

To understand the computational setting in which the contextual bias might arise, we constructed a probabilistic model of the perception of frequency shifts between tone complexes. The model took as input a noisy sequence of component tone frequencies representing the auditory stream. It then used a Factorial hidden Markov model [5], to cluster these components into tracks on the basis of their spectro-temporal continuity. Each track was defined by the mean and typical spread of frequencies with which it was associated over time. The model processed sounds in three stages, which are described below: initialization, tracking, and construction of the overall shift percept. The method to fit the model to behavioral data is then described.

Initialization

When a chord consisting of several components is input into the model, it initiates one track per component tone, but maintains uncertainty in the form of a Gaussian distribution about the central frequency of the track. The distribution is centered on the observed tone frequency and has a variance that is equal to the total variance that would be expected in the sensed frequencies of tones associated with that track. This variance is the sum of two parts: ${\sigma^2}_{\mathrm{c}} = {\sigma^2}_{\mathrm{track}} + {\sigma^2}_{\mathrm{sens}}$; the variance of acoustic frequencies associated with any one track (${\sigma^2}_{\mathrm{track}}$) and the variance of the sensory noise that corrupts the sensed frequency (${\sigma^2}_{\mathrm{sens}}$). These are the two free parameters of the model, which were estimated from the psychophysical data from several experiments, as is explained at a later stage. In order to model sensory noise reduction as a function of tone duration, for each sensed tone we computed $\sigma_{\mathrm{sens}} = \sigma_{\mathrm{sens}}\sqrt{d0/d}$ where *d*0 is a reference tone duration (150ms) and *d* is the sensed tone duration.

Tracking

As new chords are input into the model, the component tones are attributed to the different tracks, and the belief about the mean of each track is then updated to incorporate the new observed frequencies (Figure 4). Tone attribution is a "soft" process, each tone is partly attributed to all tracks with a probabilistic weighting called a "responsibility". Tracks closer in frequency to a given component tone assume greater responsibility for it. Specifically, the responsibility is given by the probability under the model that a given tone with frequency g_j might have arisen from the distribution of frequencies associated with track i. We introduce an attribution label η_j which is the (unknowable) identity of the track which actually generated tone *j*. Then the responsibility is just the probability that $\eta_j = j$. That is, if ongoing beliefs concerning the mean frequency are defined by $\{\{\mu_1, \sigma_1\}, \ldots, \{\mu_k, \sigma_k\}\}$ and the chord c is presented, we compute for each tone j and each track i, the responsibility ${r^i}_j$ as follows.

$$\mathrm{r^i}_{\mathrm{j}} = \mathrm{p}(\eta_{\mathrm{j}} = \mathrm{i}|\mathrm{g_j}, \{\mu_{\mathrm{i}}, \sigma_{\mathrm{i}}\}) \propto \mathrm{N}(\mathrm{g_j}; \mu_{\mathrm{i}}, {\sigma^2}_{\mathrm{c}})\exp(-1/2 \ * {\sigma^2}_{\mathrm{i}}/{\sigma_{\mathrm{c}}}^2)$$

Once attribution has taken place, the mean frequency of each track is updated with the frequencies all tones, weighted by the responsibilities. We update the beliefs about the ongoing tracks as follows: for each track i, we compute the effective number of tones attributed to that track, $n_i = \sum_k {r^i}_k$ and the weighted mean frequency of the tones attributed to that track, v_i.

$$v_i = 1/n_i \sum_k r^i{}_k g_k.$$

Mean and variance of the belief about track *i* are updated as follows

$$\sigma_i{}^2 \leftarrow \left(\frac{1}{\sigma^2{}_i} + \frac{n_i}{\sigma_c{}^2}\right)^{-1}$$

$$\mu_i \leftarrow \left(\frac{1}{\sigma^2{}_i} + \frac{n_i}{\sigma_c{}^2}\right)^{-1}\left(\frac{\mu_i}{\sigma^2{}_i} + \frac{v_i}{\sigma_c{}^2}\right)$$

Finally, since the prior belief about the dynamics of track means is that of a Wiener Process, variances of the beliefs about each tracks is incremented by $\gamma^2 \delta t$ where δt is the inter-onset interval between Shepard tones and γ is the assumed rate of change of track means. A wide range of values for γ led to quantitatively similar fits for slow time-scales of variation up to approximately a 10th of an octave per second. These correspond to slow variations relative to the overall duration of the context and the test pair. For this reason, and to avoid an unconstrained additional free parameter, we set this parameter to zero for all the results reported.

This process of attribution and updating is repeated for all remaining stimuli in the trial.

Shift percept construction

Finally, we modeled the behavioral response of the listener when judging the direction of pitch shift between a pair of consecutive chords. Frequency shifts were computed locally within each track, and these local shifts were then combined across tracks to build a global percept of pitch change. The local frequency shift within a track was taken to be the sum over all possible oriented shifts between pairs of consecutive tones in the two chords, weighted by how likely they were to both belong to that track.

For track *i*,

$$\phi_i = \sum_{j_1, j_2} r^i{}_{j_1} r^i{}_{j_2} (g_{j_2} - g_{j_1})$$

The global shift percept was then simply the sum of the track-local shifts:

$$\Phi = \sum_i \phi_i$$

A binary percept was constructed by thresholding Φ at 0. When Φ is positive, a rising pattern is predicted, and when Φ is negative, a falling pattern is predicted.

Fitting procedure

We generated model predictions for psychophysical experiments, where Shepard tone pairs were presented without context (Experiment 1), where one context tone was presented before the ambiguous tone pair (Experiment 2), where several context tones were presented (Experiment 2) and where the duration of one context tone was varied (Experiment 4). In order to assess the performance of the model relative to the behavioral data, we estimated the maximum-likelihood parameters of the model.

Participants performed different subsets of the full set of experiments, with four participants having completed Experiments 1, 2, and 4; seven having completed Experiments 1 and 2, and six participants having completed only Experiment 4. We took full advantage of the data available by estimating one set of the parameters, σ_{sens} and σ_{track}, for each individual using from the data from all the experiments that the listener completed. The log likelihood provided an estimate of the fit of the model predictions to the psychophysical data. The parameter σ_{track} was estimated in the range of 0.1-2 (up to two octaves) and σ_{sens} was estimated in the range of 0.01-0.5 (up to half an octave). As can be seen in Supplementary Figure 3b, the value of the parameter σ_{track} has little effect on the fit to the data up to 0.5 octaves, which is the frequency span of the context region.

Therefore, to generate the simulations shown in Figures 1 and 2, an arbitrary value in this range was selected for σ_{track}, which was fixed at 0.16 across participants, and the parameter σ_{sens} was selected to maximize the likelihood of each individual response for all tasks and conditions. An estimate of the uncertainty in the parameter value for σ_{sens} was provided by the 95% confidence interval around the mode of the normalized likelihood, displayed in Supplementary Figure 3c.

Supplementary References

1. Semal, C. & Demany, L. Individual differences in the sensitivity to pitch direction. *J. Acoust. Soc. Am.* **120,** 3907–3915 (2006).
2. Chambers, C. & Pressnitzer, D. Perceptual hysteresis in the judgment of auditory pitch shift. *Atten. Percept. Psychophys.* **76,** 1271–1279 (2014).
3. Deutsch, D., Moore, F. R. & Dolson, M. The perceived height of octave-related complexes. *J. Acoust. Soc. Am.* **80,** 1346–1353 (1986).
4. Simon, J. Z. & Wang, Y. Fully complex magnetoencephalography. *J. Neurosci. Methods* **149,** 64–73 (2005).
5. Ghahramani, Z. & Jordan, M. Factorial hidden Markov models. *Mach. Learn.* **273,** 245–273 (1997).
